# Site-selective photo-crosslinking for the characterisation of transient ubiquitin-like protein-protein interactions

**DOI:** 10.1371/journal.pone.0316321

**Published:** 2025-01-27

**Authors:** Zac Sandy, Zijuan Wang, Deepak Behera, Benjamin M. Foster, Finlay A. Martin, Kira Brüninghoff, Kathleen M. Cain, Wolfgang Dörner, Maria Jose Cabello-Lobato, Josep V. Forment, Matthew J. Cliff, Igor Larrosa, Perdita Barran, Duncan L. Smith, Henning D. Mootz, Christine K. Schmidt

**Affiliations:** 1 Manchester Cancer Research Centre, Division of Cancer Sciences, School of Medical Sciences, Faculty of Biology, Medicine and Health, University of Manchester, Manchester, United Kingdom; 2 School of Chemistry, University of Manchester, Manchester, United Kingdom; 3 Institute of Biochemistry, University of Münster, Münster, Germany; 4 Manchester Institute of Biotechnology (MIB), University of Manchester, Manchester, United Kingdom; 5 Mass Spectrometry and Separations Facility, Faculty of Science and Engineering, University of Manchester, United Kingdom; 6 Early Oncology R&D, AstraZeneca, Cambridge Biomedical Campus, Cambridge, United Kingdom; 7 Michael Barber Centre for Collaborative Mass Spectrometry, University of Manchester, Manchester, United Kingdom; 8 Cancer Research UK Manchester Institute, The University of Manchester, Manchester, United Kingdom; University of South Africa, SOUTH AFRICA

## Abstract

Non-covalent protein-protein interactions are one of the most fundamental building blocks in cellular signalling pathways. Despite this, they have been historically hard to identify using conventional methods due to their often weak and transient nature. Using genetic code expansion and incorporation of commercially available unnatural amino acids, we have developed a highly accessible method whereby interactions between biotinylated ubiquitin-like protein (UBL) probes and their binding partners can be stabilised using ultraviolet (UV) light-induced crosslinks. The stabilised protein complexes can be purified using affinity purification and identified by mass spectrometry. The resultant covalent bonds can withstand even the harshest washing conditions, allowing for the removal of indirect binders whilst retaining and capturing weak and transient interactors that are commonly lost during wash steps. This technique is widely applicable and highly effective for identifying site-selective non-covalent interactors. Members of our team have previously demonstrated the benefit of this method using the small ubiquitin-like modifier (SUMO). Here, we provide further proof-of-principle validation of the method and highlight its generality by applying an optimised workflow to a lesser studied UBL, interferon stimulated gene 15 (ISG15). We show that this method is able to capture known ISG15 interactors from a complex protein mixture in a site-selective manner, only capturing proteins that specifically interact with the region of ISG15 where the unnatural amino acid was incorporated. This exquisite degree of sensitivity and specificity greatly improves upon previous screens aimed at identifying downstream non-covalent binders, or readers, of ISG15. Taken together, the approach opens the possibility of characterising previously undetected protein-protein interactions, with the potential of elucidating molecular mechanisms behind the most complex and poorly understood processes in the cell.

## Introduction

Non-covalent, dynamic interactions underpin some of the most important processes in the cell. Indeed, over 80% of proteins have been shown to operate as part of larger protein complexes, highlighting the importance of protein-protein interactions (PPIs) [1]. However, identifying and investigating these interactions has proven to be challenging due to their weak affinity or short-lived nature. Traditional methods of screening for non-covalent PPIs often involves affinity-tag based pulldowns in which weak and transient true positive interactors can be lost due to overly stringent washing, or conversely, end up buried in a mixture of false positives due to lax conditions. Overcoming this central challenge of removing false positives while maintaining weak interactors is often a balancing act that involves multiple rounds of optimisation and rarely results in sensitive and specific enrichments of anything other than the strongest, most well-known interactors. In addition, these techniques are incapable of distinguishing between direct and indirect interactions, making the structural study of the identified protein complexes challenging. For this reason, here, we present a lab protocol for a highly specific and accessible method of stabilising these short-lived interactions using genetic code expansion and UV-induced photo-crosslinking based on site-selective integration of an unnatural amino acid containing benzophenone, one of the most commonly used and studied photo-crosslinking moieties [2–5]. This can then be followed by streptavidin pulldowns and mass spectrometry (MS) to identify novel interactors that have been covalently captured.

The initial development of the method was carried out using the small ubiquitin-like modifier SUMO, and identifying protein-protein interactions it can mediate [6–8]. Similar to ubiquitin, ubiquitin-like proteins (UBLs) like SUMO act as a form of post-translational modification and play crucial roles in various cellular processes such as protein degradation, the immune response, autophagy, transcription, and DNA repair [9–11]. By covalently modifying substrate proteins in a process known as UBLylation, UBLs such as SUMO and interferon stimulated gene 15 (ISG15) are capable of altering the activity, localisation, stability, and interactomes of modified substrates. Importantly, these UBL marks can also be read by receptor proteins that dynamically bind in a non-covalent fashion with varying functional consequences [12–15]. Unlike UBL substrates, these readers of UBLs and UBL marks can often bind weakly or transiently, making them challenging to identify, despite their critical importance. However, as UBLs are generally well defined structurally and contain suspected or known binding hotspots, they are ideal candidates for the method described herein. Indeed, members of our team have recently shown this to be a powerful technique in the exploration of non-covalent binders of SUMO [6–8]. In these studies, numerous potential interactors of SUMO proteins were identified in a site-selective manner, with previously validated non-covalent interactors of SUMO such as ubiquitin activating enzyme 2 (UBA2), Ran binding protein 2 (RanBP2), protein inhibitor of activated STAT1 (PIAS) and receptor associated protein 80 (RAP80) being robustly identified [7]. In addition, a novel SUMO interacting motif (SIM) was identified in the DNA damage response protein MRE11, highlighting the potential promise of this technique to uncover novel molecular mechanisms and pathways [7]. Excitingly, the strategic placement of the photo-crosslinking moiety enabled the identification of binding partners in a regioselective fashion, with the technique facilitating differential discovery of class I and class II SUMO interactors by SUMO1 probes integrating the photo-crosslinkable BpF in distinct interaction regions [8].

In this protocol, we further develop this method in terms of understanding the underlying design rules and exploring its generality when applied to other protein types. To this end, we use the UBL ISG15, because identifying protein interactors of this related, yet distinct posttranslational modifier represents a similar challenge. Being able to capture ISG15 binding partners thus serves as a further demonstrative model for this platform. Structurally, ISG15 is 15 kDa in size and resembles a UBL dimer with two ubiquitin-like domains joined together by a flexible linker region [16]. Like other UBLs, ISG15 is capable of covalently modifying substrate proteins in a process called ISGylation, which plays various roles in innate immunity, protein homeostasis, and genome stability [17–19]. However, compared to SUMO, relatively few non-covalent binders of ISG15 have been identified, possibly suggesting that the ISG15 interactome consists of weaker, more transient interactions. Despite this, recent identification of non-covalent ISG15 ‘reader’ proteins have become fundamental to our understanding of how ISG15 functions mechanistically. For example, while ISG15 was long understood to target newly transcribed, potentially suspicious proteins during viral infection, only recently was it discovered that RING finger protein 213 (RNF213) can act as a sensor for these marks, binding non-covalently to sequester ISG15-tagged viral proteins [15]. Similarly, the identification of the cell surface receptor lymphocyte function-associated antigen 1 (LFA1) as an ISG15-binding protein led to the understanding that free ISG15 can act as an extracellular signalling molecule with cytokine-like properties [13]. ISG15 reader proteins have also been discovered outside of an innate immune context, with the recent discovery of the RecQ-like helicase, RECQ1, as an ISG15 binder, being the first of a series of recent studies highlighting novel roles for ISG15 at the DNA replication fork [14, 20, 21].

Taken together, these studies highlight the exciting and demonstrated ability of the identification of reader proteins of different UBLs using a highly sensitive screening method. In addition, the photo-crosslinking approach presented here has the potential to stratify ISG15 interactors by where precisely on ISG15 they bind. Insights into binders of the two different UBL domains of ISG15 may shed light on any potentially distinct functions of the domains, which is currently an open question in the field. Moreover, by demonstrating the utility of this method using different UBLs [6–8, 22], we foresee this method being widely applicable, including in the growing field of PPIs mediated by intrinsically disordered regions (IDRs).

## Materials and methods

The protocol described in this peer-reviewed article is published on protocols.io (dx.doi.org/10.17504/protocols.io.5qpvo9yqbv4o/v3) and is included for printing purposes as S1 File.

**Fig 1:**
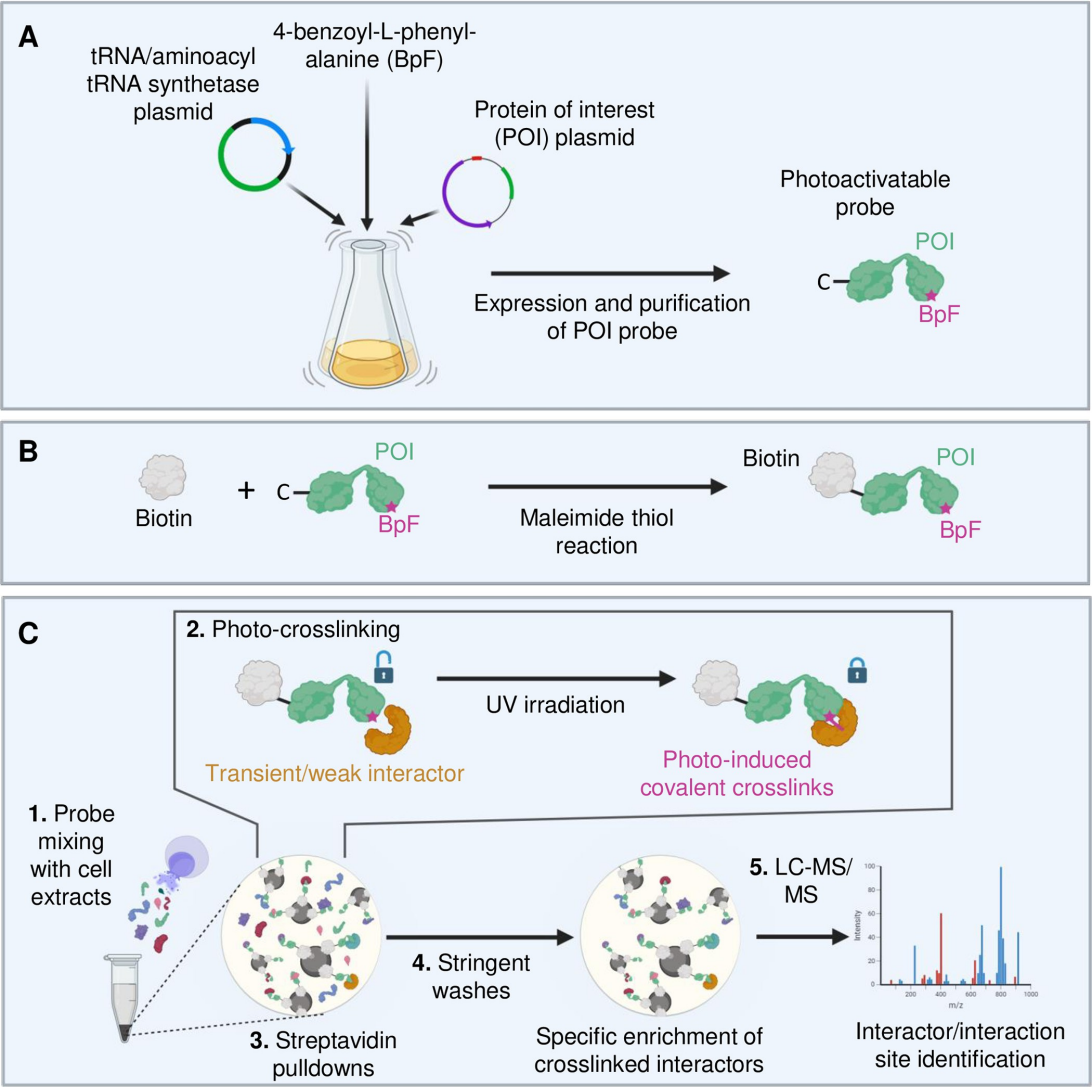
Experimental pipeline for protein-protein interaction identification and characterisation using photoactivatable crosslinking. (**A**) Generation of recombinant probes of the protein-of-interest: a plasmid containing the desired protein probe is mutated to contain both an amber stop codon in a region of interest and a cysteine for downstream biotinylation (see (B) for details regarding biotinylation). For the probe, the genetic code is expanded by co-transforming *Escherichia coli* with plasmids encoding the engineered protein-of-interest and an established bio-orthogonal tRNA/aminoacyl-tRNA synthetase pair that recognises the amber stop codon and incorporates the unnatural amino acid 4-benzoyl-(L)-phenylalanine (BpF) into the amber position. (**B**) Maleimide chemistry using a commercially available kit (Sigma, #cat21901BID) to bioconjugate a single biotin molecule to a cysteine (C) placed at one of the termini of the probe (see (A) above). Endogenous surface-accessible cysteines are mutated to alanine or serine residues to prevent attachment of multiple biotin molecules. (**C**) 1. The probes are combined with simple or complex protein mixtures (e.g. cellular extracts); 2. The probes combined with the protein mixtures are subjected to a defined ultraviolet (UV)-irradiation step, using a commercially available hand-held lamp, to probe transient and weak interactors. 3. UV-crosslinked interactors are enriched via the biotinylated probe using streptavidin beads and 4. subjected to stringent wash steps to remove indirect and non-specific binders prior to downstream analysis. 5. The identification of the covalently crosslinked interactors can be performed using immunoblotting or combined with liquid chromatography tandem mass spectrometry (LC-MS/MS). Created in BioRender. Foster, B. (2025) https://BioRender.com/w84q853.

Briefly, in a first instance, we generated photo-crosslinking mutants of the protein-of-interest (POI) to include both a terminal cysteine and an amber stop codon in the suspected site of interaction. This was then followed by co-transforming *Escherichia coli* with a plasmid encoding the engineered protein-of-interest and an established bio-orthogonal tRNA/aminoacyl-tRNA synthetase pair that can recognise the amber stop codon and facilitate the incorporation of the unnatural photo-activatable amino acid 4-benzoyl-(L)-phenylalanine (BpF) into the amber position (Fig 1A) [23]. This unnatural amino acid is particularly advantageous as it is commercially available, well tolerated by bacterial cells, and often incorporated with high efficiency.

One of the key features of this method is an integrated biotinylation step (Fig 1B). For this, we used maleimide chemistry to bioconjugate a single biotin molecule to a cysteine placed in the N- or C-terminus of the probe. However, adding a cysteine as the second amino acid after methionine should be avoided, given that methionines are often cleaved off, which would leave the protein with an extreme N-terminal cysteine. Such cysteines, constituting a free 1,2-amino-thiol group, could react with other molecules in the cell, for example aldehydes and pyruvate [24], which would prevent the thiol from conjugating with biotin. Therefore, especially for larger proteins, it is worth positioning the cysteine towards the C-terminus to avoid the pulldown of biotin-tagged protein fragments, truncated at the amber stop codon due to incomplete integration of the unnatural amino acid. However, this would depend on the specific properties of the engineered protein-of-interest and the efficiency of its translation. If the probe contains endogenous surface-accessible cysteines, their mutation to alanines or serines to prevent attachment of multiple biotin molecules is recommended. If cysteine modification is not plausible, other methods for bioconjugation such as sortase A-mediated modification of a short tag peptide [25] or split-intein-mediated chemical modification [26] could be explored.

To induce photo-crosslinking, we combined the probes with simple or complex protein mixtures (Fig 1C, step 1) and subjected them to UV-irradiation using a commercially available and affordable hand-held lamp (Fig 1C, step 2). This approach facilitates the capture of interacting proteins in a highly regio-selective manner as only proteins captured in close proximity to the incorporated BpF were crosslinked [8]. We then employed streptavidin pulldowns using harsh, denaturing conditions due to the addition of the biotin-tag (Fig 1C, steps 3 and 4), enabling the resultant proteins to be identified using immunoblotting and mass spectrometry methods (Fig 1C, step 5).

## Expected results

**Fig 2:**
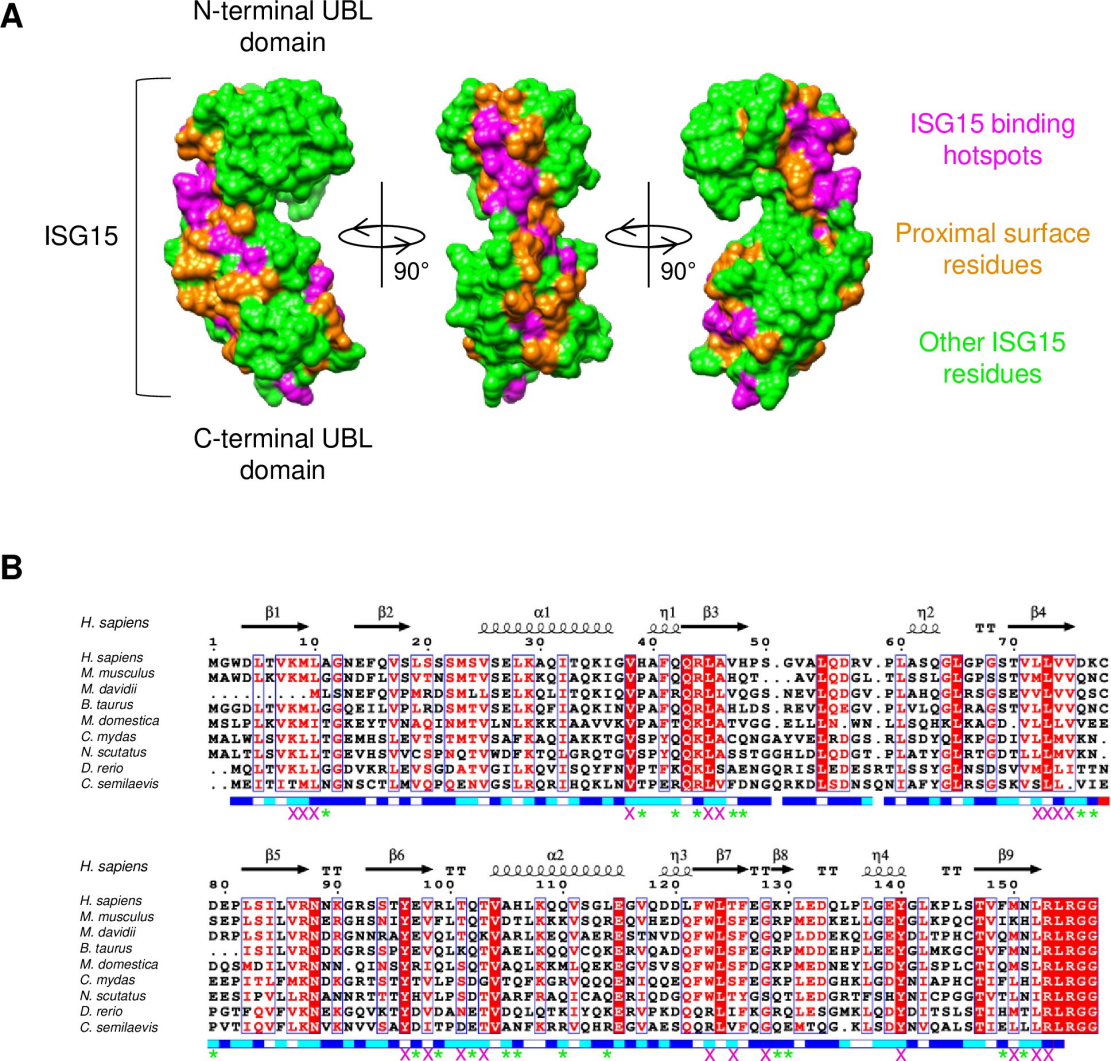
Selection of candidate sites for integrating photo-crosslinkable residues. (**A**) Non-covalent binding hotspots of ISG15 (PDB: 1Z2M; magenta) were identified using crystal structures and information available from the literature. Surface-accessible amino acids proximal to these sites are highlighted in orange, the remaining ISG15 residues in green. (**B**) Multiple sequence alignment of the indicated ISG15 orthologues. Red lettering and blue boxes represent conserved residues (>70%), considering physicochemical properties of residues, whereas red boxes represent residues conserved across all species (100%). Secondary structure (α: alpha helix; η: 310 helix; β: beta-strand; TT: beta-turn) and predicted solvent accessibility (dark blue: accessible; light blue: intermediate; white: buried) of human ISG15 (PDB: 1Z2M) is detailed above and below. Magenta crosses beneath amino acids represent residues excluded from consideration due to high conservation whereas green asterisks represent residues deemed suitable for potential mutation into amber stop codons to integrate the photo-crosslinkable unnatural amino acid 4-benzoyl-(L)-phenylalanine (BpF).

To begin developing a photo-crosslinking screening platform for a new protein-of-interest like ISG15, the intended sites of incorporation for BpF had to be selected. The reported radius for BpF crosslinking after UV photoactivation is between 3.1 and 20 Å [27–29] and, while this suggested that crosslinking would be highly selective, it also meant that the site of incorporation for BpF must be carefully considered as this could hamper the number of interactors captured if placed too distal from protein-protein interaction sites. To this end, we compiled a list of binding regions on ISG15 using the limited information available in the literature [13, 14, 30–39]. From this list, three hotspots were identified: an N-terminal hydrophobic patch similar to ubiquitin, a small patch in the hinge region reported to be important for LFA1 binding, and a C-terminal patch associated with the binding of various proteases. Structural analysis was used to identify proximal, solvent-accessible amino acids as potential candidates for mutation into BpF (Fig 2A, residues highlighted in orange). These sites were then further triaged using conservation analysis, excluding any mutation candidate that was highly conserved across species as these residues were more likely to be crucial for structural stability (Fig 2B). In addition, preference was given for arginine and phenylalanine mutations, the former having been shown to be particularly amenable to BpF mutation in previous studies [6–8, 22], and the latter being an amino acid similar to BpF, therefore making its mutation less likely to cause structural disruptions. While limited sites were available for ISG15, tyrosines represent alternative target residues for mutation as they are commonly surface-facing with protruding side chains and situated at protein-protein interaction hotspots [40]. A total of 7 amino acids were chosen as mutation candidates and the recombinant probes purified, together representing the multiple binding hotspots of ISG15. As ISG15 contains a single internal cysteine at position 78, all crosslinking probes contained a C78S mutation, a modification previously reported to be well-tolerated in regard to ISG15 stability and function [16, 41], with an additional cysteine being included at the C-terminus for downstream maleimide-mediated linking to biotin.

**Fig 3:**
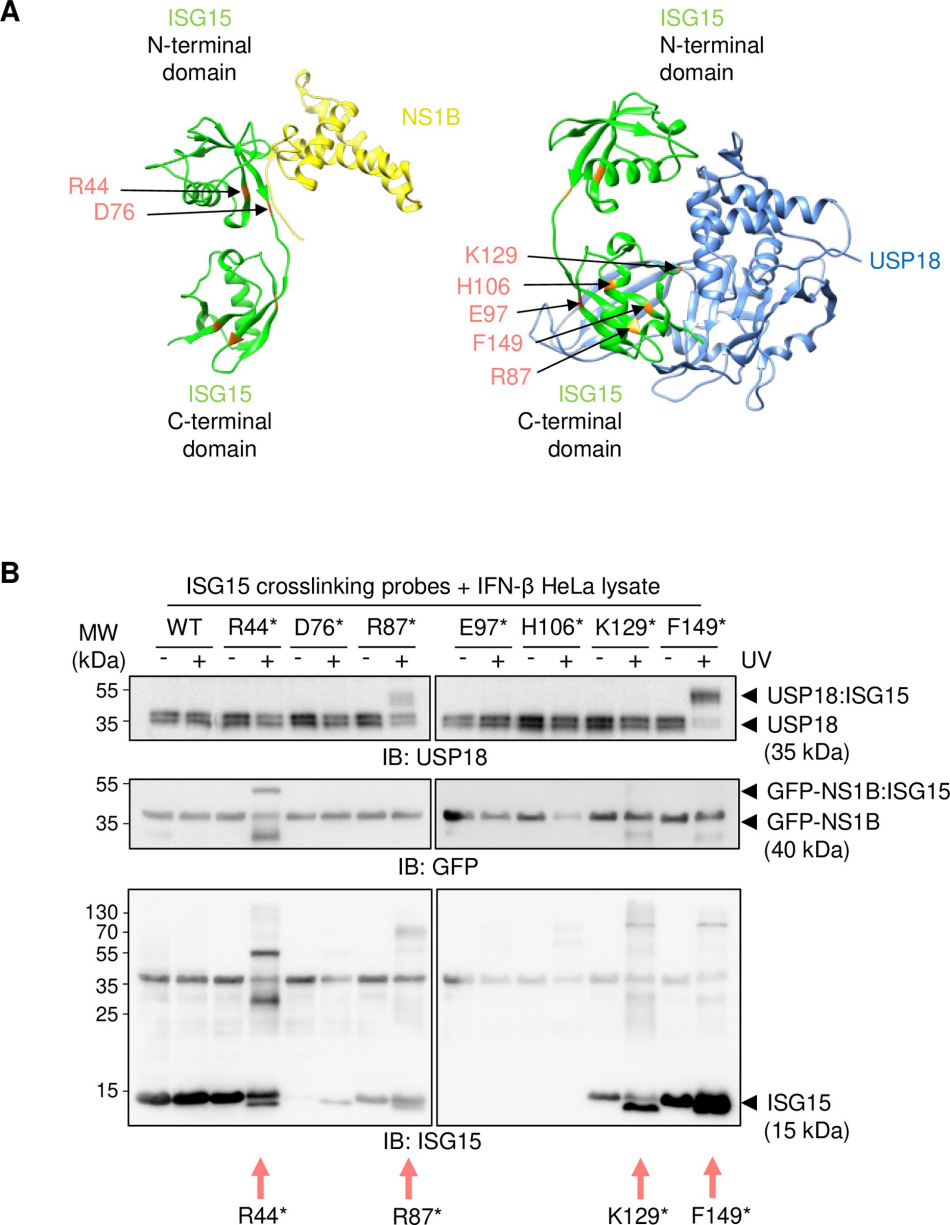
Site-specific photo-crosslinking between ISG15 and known non-covalent interactors. (**A**) Overlayed structures of the interaction between ISG15 (green) and Usp18 (blue; PDB: 5CHV) and the N-terminal region (residues 1–103) of NS1B (yellow; PDB: 3SDL). Note that as structures are not available for human USP18, the crystal structure of interacting mouse Usp18 and Isg15 was used as a template and overlayed with human ISG15 (PDB: 3SDL). For NS1B, the other half of the NS1B homodimer was excluded for clarity as it does not interact with the same ISG15 molecule. (**B**) Photo-crosslinking assay using 20 μM of the recombinant ISG15 probes containing the photo-crosslinkable unnatural amino acid 4-benzoyl-(L)-phenylalanine (BpF) at the indicated sites. Photo-crosslinkable ISG15 probes are indicated with asterisks following the residue where the BpF was integrated. Wild-type (WT) ISG15 was used as a control. All recombinant proteins were mutated to replace cysteine 78 with serine and integrate a C-terminal cysteine for downstream biotinylation. Probes were mixed with lysate generated from HeLa cells that had been transfected with GFP-NS1B (residues 1–103) and treated with 100 U/mL IFNβ for 24 hours to induce *USP18* expression. Crosslinking was confirmed by the appearance of a 15 kDa band shift in USP18 and GFP immunoblots (IBs). Red arrows indicate ISG15 probes that successfully formed crosslinks in response to UV as indicated by a laddering pattern in the ISG15 immunoblot. MW: molecular weight.

To test whether these purified photo-crosslinking probes were capable of specific UV crosslinking, we used the known ISG15 interactors USP18 and NS1B as positive controls. USP18 is an interferon-stimulated gene and canonical deISGylase whereas NS1B is a viral protein reported to strongly bind to ISG15 [30, 39]. These were chosen, as each interacted with a different UBL domain of the ISG15 molecule, allowing us to assess whether the probes were site-specific (Fig 3A). The ISG15 probes were mixed with lysate prepared from HeLa cells, ectopically expressing GFP-NS1B, and IFNβ-treated to induce endogenous USP18 expression before being treated with UV light for 2 hours and subsequently subjected to analysis. Immunoblotting revealed that not all probes were capable of photo-crosslinking or staying stable in solution, with only 4 out of the 7 probes showing UV-induced laddering in the ISG15 blot, indicative of multiple ISG15-crosslinked proteins at various molecular weights (Fig 3B). Importantly, this UV-induced laddering was not present when wild-type (WT) ISG15 was treated with UV light, precluding the possibility of endogenous, UV-induced crosslinks with ISG15. Most exciting, however, was that crosslinking of the positive controls was restricted to mutants with crosslinking residues in close proximity to the interaction site. When blotting for USP18, clear UV-induced, ~15 kDa band shifts were detectable for the ISG15 R87 and F149 BpF probes, indicated in the following as R87* and F149*, in line with crosslinking of USP18 with ISG15 (Fig 3B). Of the four probes capable of crosslinking (R44*, R98*, K129* and F149*), these were the two most associated with the USP18 interaction site. Strikingly, the K129* probe, despite being capable of photo-crosslinking, did not form detectable crosslinks with USP18, with the K129 residue being more distant compared to R87 and F149, but still relatively proximal, to the USP18 interaction site (Fig 3A, right). Similarly, the R44* probe was the only crosslinkable ISG15 probe that produced a UV-induced 15 kDa band shift for GFP-NS1B, as was predicted structurally. Taken together, these findings highlight the high regioselectivity of the approach, with no published ISG15 screening method having achieved this level of specificity to date.

**Fig 4:**
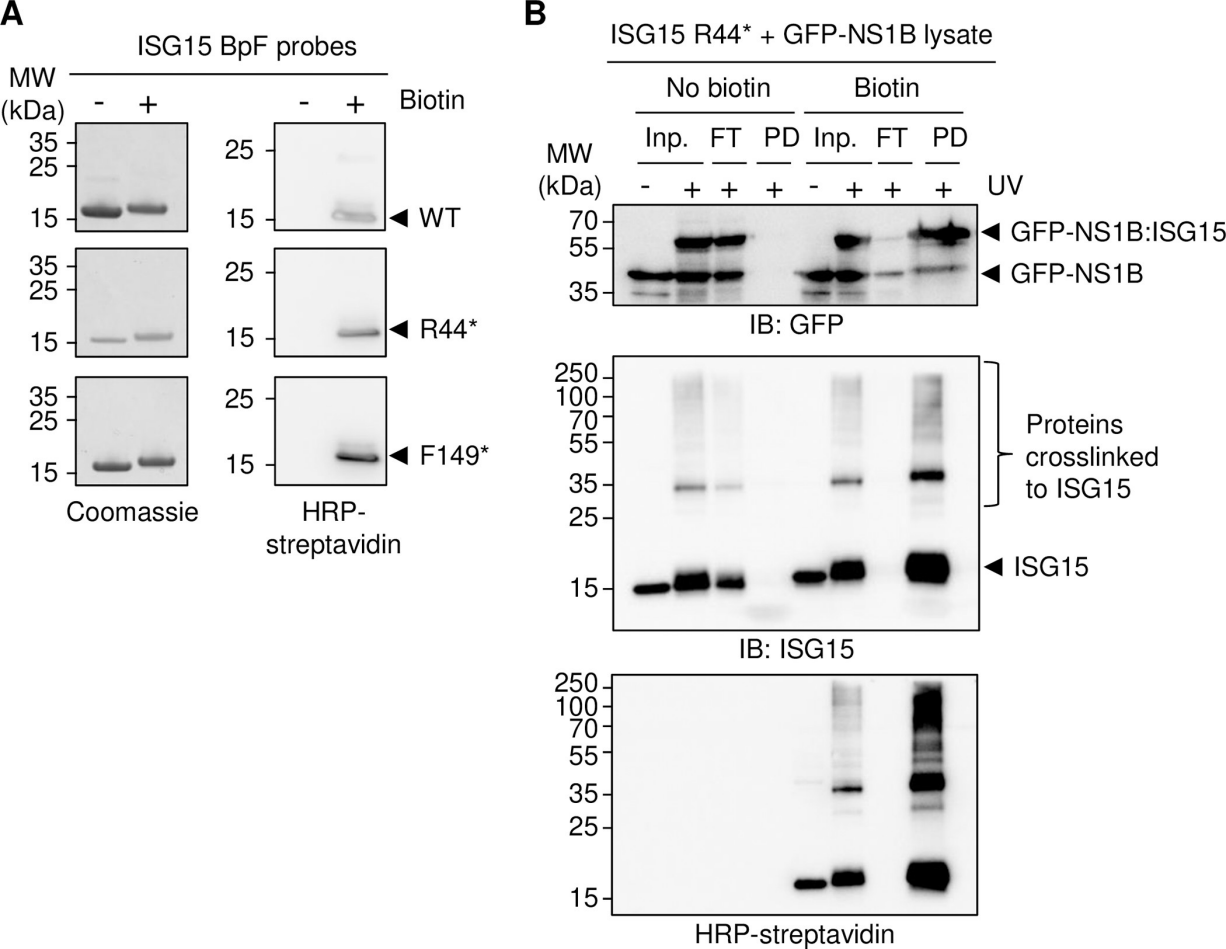
Biotinylation, pulldown, and elution of crosslinked ISG15 interactors. (**A**) Recombinant ISG15 proteins, wild-type (WT) or integrating the unnatural amino acid 4-benzoyl-(L)-phenylalanine (BpF) at positions arginine R44 and phenylalanine F149 (R44* and F149*), biotinylated at a unique cysteine introduced to the C-terminus (the endogenous cysteine at residue 78 was replaced with serine) were loaded and stained with Coomassie brilliant blue (left, 4–12% Bis-Tris PAGE in MES running buffer) or blotted to detect the biotinylated proteins using streptavidin conjugated to HRP (right, homemade 15% SDS-PAGE in Tris-glycine running buffer). (**B**) Photo-crosslinking assay using a biotinylated and non-biotinylated recombinant ISG15 C78S probe containing BpF at residue R44 (R44*). Probes were mixed with lysate generated from HEK293T cells, transfected with a GFP-tagged N-terminal domain of the ISG15 interactor NS1B and treated with ultra-violet light (UV) for 2 hours. Streptavidin pulldowns (PDs) were assessed using immunoblotting. 5% (v/v) of the input (Inp.) and flow-through (FT) were loaded. IB: immunoblot; MW: molecular weight.

As mentioned above, a unique feature of this method as opposed to previously published photo-crosslinking protocols is the addition of a biotin tag. The extremely high affinity of the biotin-avidin interaction and its high resistance towards chemical denaturants such as urea provided by this tag facilitates intensely stringent washing conditions aimed at removing non-specific or indirect binders. As such, we biotinylated the R44* ISG15 probe using a commercially available maleimide-reaction kit targeting the cysteine incorporated at the C-terminus of the recombinant probes. This prevented multiple biotin molecules being added to ISG15 and potentially sterically hindering the binding of non-covalent interactors. Successful biotinylation was monitored by western blotting using streptavidin coupled to horse radish peroxidase (HRP) (Fig 4A). We next carried out streptavidin pulldowns under extensive wash conditions following capture of the biotinylated ISG15 probe. This consisted of a total of 35 washes in 8 M urea with high salt under both low and high pH conditions. Using the R44* probe as a model, we were able to capture a large number of UV-induced ISG15-crosslinked proteins, with no or minor loss of proteins detectable in the flow-through (FT). These findings indicate that various proteins interacted and crosslinked with the ISG15 probe, which are amenable to identification by mass spectrometry, similar to what members of our team have previously demonstrated for photo-crosslinkable probes based on SUMO [7, 8]. As expected, crosslinked GFP-NS1B was enriched amongst the pulled-down proteins (Fig 4B). Taken together, the highly sensitive and specific nature of these pulldowns paired with the regioselectivity of the UV-induced photo-crosslinking emphasize the capability of this method of overcoming the typical challenges faced by traditional screening methods. Given the promise of this technique, proteomics studies following these pulldowns have potential to provide vital insights into the elusive and hard-to-define ISG15 interactomes. Such databases can serve as a platform for identifying ISG15-regulated cell signalling pathways in health and disease, and have potential to be combined with additional downstream investigations such as structural characterisation.

## Conclusions and future perspectives

While we chose to demonstrate the feasibility and promise of the approach using the UBLs SUMO and ISG15, it is noteworthy that the method has potential to be invaluable beyond ubiquitin/UBLs. For instance, this technique could prove especially useful when investigating binding interfaces involving IDRs of proteins. These regions are increasingly being recognised for their ubiquitous nature and physiological functionality, yet the majority of them remain poorly understood. Critically, their significance is thought to be mediated substantially by PPIs. However, due to the short-lived and variable nature of IDR-mediated PPIs, these interactions have been notoriously hard to study using conventional methods. IDRs are common, with numerous cell signalling pathways relying on IDR-containing proteins (e.g. MYC, p53 and TAU), which often represent attractive, yet unexploited, drug targets [42]. Our approach provides an attractive platform for addressing key limitations linked to PPI discovery in these areas. In the longer term, the method therefore has potential to contribute to the development of more targeted precision medicines in a wide range of diseases.

## Supporting information

S1 File Step-by-step protocol. This file is also available on protocols.io.
(PDF)

S1 Raw images Raw blot and gel images. (PDF)
