# Supplementary material for: Site-selective photo-crosslinking for the characterisation of transient ubiquitin-like protein-protein interactions
Source: PLoS One. 2025 Jan 27;20(1):e0316321. doi: 10.1371/journal.pone.0316321 (PMC11771908; doi:10.1371/journal.pone.0316321)
Supplement: S1 File — This file is also available on protocols.io. (PDF) [file pone.0316321.s001.pdf]

Nov 27, 2024 Version 3

Site-specific incorporation of BpF into ubiquitin-like proteins (UBLs) in *E. coli* V.3

DOI
dx.doi.org/10.17504/protocols.io.5qpvo9yqbv4o/v3

Zac Sandy[1], Zijuan Wang[1], Deepak Behera[1,2], Benjamin M Foster[1], Finlay A Martin[1], Kira Brüninghoff[3], Wolfgang Dörner[3], Kathleen M Cain[4], Maria Jose Cabello-Lobato[1], Josep V Forment[5], Matthew Cliff[2,6], Igor Larrosa[2], Perdita Barran[2,4,6], Duncan L Smith[7], Henning D Mootz[3], Christine K Schmidt[1]

[1]Manchester Cancer Research Centre, Division of Cancer Sciences, School of Medical Sciences, Faculty of Biology, Medicine and Health, University of Manchester, Manchester, UK;
[2]School of Chemistry, University of Manchester, Manchester, UK;
[3]Institute of Biochemistry, University of Münster, Münster, Germany;
[4]Michael Barber Centre for Collaborative Mass Spectrometry, University of Manchester, Manchester, UK;
[5]Early Oncology R&D, AstraZeneca, Cambridge Biomedical Campus, Cambridge, UK;
[6]Manchester Institute of Biotechnology (MIB), University of Manchester, Manchester, UK;
[7]Cancer Research UK Manchester Institute, University of Manchester, Manchester, UK

Benjamin Foster
University of Manchester

OPEN ACCESS

DOI: dx.doi.org/10.17504/protocols.io.5qpvo9yqbv4o/v3

Protocol Citation: Zac Sandy, Zijuan Wang, Deepak Behera, Benjamin M Foster, Finlay A Martin, Kira Brüninghoff, Wolfgang Dörner, Kathleen M Cain, Maria Jose Cabello-Lobato, Josep V Forment, Matthew Cliff, Igor Larrosa, Perdita Barran, Duncan L Smith, Henning D Mootz, Christine K Schmidt 2024. Site-specific incorporation of BpF into ubiquitin-like proteins (UBLs) in E. coli. **protocols.io** **https://dx.doi.org/10.17504/protocols.io.5qpvo9yqbv4o/v3**Version created by **Benjamin Foster**

License: This is an open access protocol distributed under the terms of the **Creative Commons Attribution License**, which permits unrestricted use, distribution, and reproduction in any medium, provided the original author and source are credited

Protocol status: Working
We use this protocol and it's working

Created: November 11, 2024

Last Modified: November 27, 2024

Protocol Integer ID: 113010

Keywords: Ubiquitin-like proteins (UBLs), Escherichia coli, BpF-incorporated protein

Funders Acknowledgement:
MRC research grant
Grant ID: MR/X008754/1
David Phillips Fellowship
Grant ID: BB/N019997/1
Deutsche Forschungsgemeinschaft
Grant ID: SFB858/B-14

Abstract

This protocol details the site-specific incorporation of BpF into ubiquitin-like proteins (UBLs) in *E. coli*. The use of a photo-crosslinkable residue within UBLs enhances the detection of weak and transient protein-protein interactions. This protocol provides a general workflow for the production of such protein probes, followed by methods to detect specific protein interactions using targeted or proteomic approaches. This protocol is linked to the PLOS ONE protocols manuscript "Site-selective photo-crosslinking for the characterisation of transient ubiquitin-like protein-protein interactions".

Materials

1 × SDS sample buffer

A	B
Tris-HCl pH 6.8	60 mM
SDS	2%
glycerol	10%
bromophenol blue	0.01%
beta-mercaptoethanol	1%

Prescission protease buffer

A	B
Tris-HCl pH 7.5	50 mM
NaCl	150 mM
EDTA	1 mM
DTT	1 mM

Protease mix

A	B
Tris-HCl pH 7.5	50 mM
NaCl	150 mM
EDTA	1 mM
DTT	1 mM

Buffer A

A	B
urea	8 M
SDS	2%
Tris	100 mM
NaCl	200 mM
pH 8	adjusted with HCl

Buffer B

A	B
urea	8 M
SDS	0.2%
ethanol	10%
isopropanol	10%
Tris	100 mM
NaCl	1.2 M

A	B
pH 8	adjusted with HCl

Buffer C

A	B
urea	8 M
SDS	0.2%
ethanol	10%
isopropanol	10%
Tris	100 mM
NaCl	200 mM
pH 5	adjusted with HCl

4. **Buffer D**

A	B
urea	8 M
SDS	0.2%
ethanol	10%
isopropanol	10%
Tris	100 mM
NaCl	200 mM
pH 9	adjusted with HCl

pEVOL-pBpF **addgene Catalog #**31190

EZ-Link™ Maleimide-PEG2-Biotin **Thermo Fisher Catalog #**21901BID

Zeba™ Spin Desalting Columns, 7K MWCO, 0.5 mL **Thermo Fisher Catalog #**89883

Streptavidin Sepharose High Performance, 5 mL **Cytiva Catalog #**17511301

spin columns (Pierce Micro-Spin Columns, 10510824)

Triethylammonium bicarbonate (TEAB) **Merck MilliporeSigma (Sigma-Aldrich) Catalog #**T7408

20 µL PreScission Protease (Cytiva, # Prescission Protease **Genscript Catalog #**Z02799), or suitable alternative

Transformation of *E. coli* with plasmids encoding the BpF-aminoacyl-tRNA synthetase/tRNA pair and the amber mutant of the UBL to express the BpF-incorporated protein

23h 43m

1 Mix 25 µL of *E. coli* (BL21(DE3) or similar expression strain that does not have chloramphenicol resistance) chemically competent cells with 50 ng – 100 ng of pEVOL-pBpF (Addgene #31190) and 50–100 ng of an expression plasmid containing the protein of interest with the TAG amber codon for incorporating the unnatural photo-activatable amino acid 4-benzoyl-(L)-phenylalanine (BpF), in a microcentrifuge tube. Incubate On ice for 00:30:00 .

30m

Note

pEVOL-pBpF was a gift from Peter Schultz (Addgene plasmid #31190; RRID:Addgene_31190). Human ISG15 (uniprot ID: P05161) was expressed as the mature form (amino acids 2-157) with an additional C-terminal cysteine before the non-amber STOP codon to enable biotinylation. ISG15 cDNA was inserted into a pGEX-6P1 plasmid backbone using BamHI and XhoI restriction sites to have an N-terminal GST-3C tag. The C78S mutation and amber STOP (TAG) codons were prepared by site-directed mutagenesis. Plasmid maps and sequences are available upon request.

2 Heat-shock the *E. coli*-plasmid DNA mixture by placing into a 42 °C water bath for 00:00:30 and then incubate On ice for 00:01:00 - 00:02:00 .

3m

3 Add 700 µL LB broth or SOC media and incubate at 37 °C for 01:00:00 while shaking.

1h

4 Plate 100 µL - 200 µL of the competent cells onto an LB agar plate supplemented with 50 undetermined ampicillin and 34 undetermined chloramphenicol.

- Incubate the LB agar plates at 37 °C Overnight .

1h

5 The next day, grow a single transformed colony in 5 mL - 25 mL of LB broth supplemented with 50 undetermined ampicillin and 34 undetermined chloramphenicol at 37 °C Overnight with shaking.

1h

6 Inoculate the overnight bacterial culture with ratio 1 in 100 into a 2 L flask and grow the *E. coli* culture for ~ 02:00:00 at 37 °C until the OD_{600} value reaches ~0.6, as monitored by a spectrophotometer.

2h

Note

Dissolve the unnatural amino acid photo-crosslinker (4-benzoyl-L-phenylalanine, BpF, CAS number: 104504-45-2) to 334 undetermined (e.g. 0.27 g in 3 mL for a 500 ml LB volume) in [M] 1 Molarity (M) NaOH solution while the culture is growing.

7 Once the OD_{600} value reaches ~0.6, add 0.05% (w/v) arabinose, [M] 2 millimolar (mM) BpF (v/v) and [M] 200 micromolar (µM) IPTG as final concentration to induce the expression of the engineered UBL probe. Transfer the bacterial suspension to 20 °C for a further 12:00:00 - 16:00:00 . 18h

Note

Expression conditions such as IPTG concentration, temperature, length of time can be optimised for the protein of interest.

8 Harvest the bacteria by centrifuging at 4.000 x g, 4°C, 00:10:00 and discard the supernatant. The pellet can be stored at -70 °C or taken forward for lysis and downstream purification. 10m

Check protein expression 6m

9 Centrifuge 1 mL of *E. coli* culture grown from before and ~ 250 µL (approximately similar number of cells following overnight growth) of *E. coli* culture from after expression at 13000 x g, Room temperature, 00:01:00 . 6m

- Discard the supernatants and suspend the pellets with 100 µL of 1 × SDS sample buffer. Heat the samples at 95 °C for 00:05:00 .

1 × SDS sample buffer

A	B
Tris-HCl pH 6.8	60 mM
SDS	2%
glycerol	10%
bromophenol blue	0.01%
beta-mercaptoethanol	1%

10 Load 5 µL of each sample onto a denaturing SDS-PAGE gel (homemade, percentage depends on predicted molecular weight (MW) of the protein of interest) and analyse by staining

with Coomassie brilliant blue or Western blot if necessary.

Protein purification (GST affinity purification) and biotinylation

34m 30s

11

Note

Protein of interest purification can be achieved using any affinity or other approaches with additional downstream purification steps, if required. The example described here is for a GST-tagged protein expressed using the pGEX-6P1 backbone plasmid followed by tag removal with 3C/Prescission protease.

Re-suspend the bacterial pellet in 5x pellet volumes of lysis buffer (e.g. if the pellet weighs 1 g, use 5 mL lysis/binding buffer). Lysis buffer in this example is PBS pH 7.4 supplemented with [M] 1 millimolar (mM) DTT and 1x protease inhibitors.

12 Sonicate the pellet On ice at 40% amplitude (QSonica Q125 sonicator with a 3.2 mm diameter probe) for 00:04:00 total time with a 00:00:30 on, 00:00:30 off, cycle to lyse the cells. 4m

13 Clarify the lysate by centrifugation at 20000 x g, 4°C, 00:30:00 to pellet insoluble material and filter the supernatant through a 0.45 µm syringe filter to remove cellular debris. 30m

14 For a 1 mL GSTrap column (Cytiva), wash the column with 5 column volumes (CV) water using a 1 mL/min flow rate. Equilibrate the column with binding buffer by washing through 10 CV of cold buffer at a 1 mL/min flow rate.

15 Inject the lysate onto the column at a slow flow rate (~0.2-0.5 mL/min) and collect the flow-through.

16 Wash the column with 10 mL (10 CV) of binding buffer at a flow rate of 1 mL/min.

17 Equilibrate the column with 10 mL of Prescission protease buffer at a rate of 1 mL/min.

Prescission protease buffer

A	B
Tris-HCl pH 7.5	50 mM
NaCl	150 mM
EDTA	1 mM
DTT	1 mM

18 Use a syringe adaptor to load 1 mL of protease mix onto the column and incubate at 4 °C Overnight to remove the GST-tag. 30s

Protease mix

A	B
Tris-HCl pH 7.5	50 mM
NaCl	150 mM
EDTA	1 mM
DTT	1 mM

- PreScission protease (Cytiva, #Z02799) or suitable alternative

19 Collect the flow-through (containing untagged protein of interest) with 3 mL of Prescission protease buffer.

20 Run samples of the lysate input and the eluted fractions on an SDS-PAGE gel to check for the yield and purity of the protein of interest.

Biotinylation of recombinant UBL probes

1h

21 Biotinylation of the engineered cysteine was carried out using an EZ-Link biotin maleimide reaction kit (Sigma, #cat21901BID).

22 An overnight incubation was carried out according to the manufacturer's instructions. Excess biotin was removed using 7 undetermined MWCO Zeba spin desalting columns (ThermoScientific, #cat89883) equilibrated with PBS.

23 Another shorter 01:00:00 incubation was carried out according to the manufacturer's instructions. Following the second incubation, excess biotin was again removed using 7 kDa MWCO Zeba spin desalting columns (ThermoScientific, #cat89883) equilibrated with PBS. 1h

24 Successful biotinylation can be discerned by Western blot using streptavidin-HRP, affinity purifications using Streptavidin beads (see below), or by intact-mass spectrometry of the probe.

Photo-induced crosslinking with purified, recombinant UBL probes and binding partners

1h 20m

25 Incubate the BpF-containing UBL (a final concentration of [M] 20 micromolar (µM) is a suitable starting point, followed by optimisation depending on the specific properties of the interaction between the UBL and its binding partner) with the respective binding partner (10 µM 15m

or 20 µM or optimised depending on affinity for the UBL) in PBS buffer for 00:15:00 at 4 °C in 0.2 mL thin-walled polypropylene PCR tubes, with a maximum volume of 50 µL - 100 µL .

26 Divide the sample into two equal volumes, with half incubated at Room temperature , and the other half irradiated for 01:00:00 with long-wave UV light (λ=365 nm; Herolab UV-16 L, 6 W, 6 mm distance). 1h

Note

Photo-crosslinking time and temperature can be optimised depending on the protein of interest and target. The irradiated sample will be warmed by approximately 3 °C during incubation, but this does not lead to any visible precipitation in our experiments.

27 Following irradiation, add an equal volume of 2 × SDS sample buffer followed by heating for 00:05:00 at 95 °C . 5m

- Check the crosslinked band by running an SDS-PAGE gel.
- The covalent bond remains under denaturing conditions and a band shift of the crosslinked proteins can be visualised by Coomassie-staining or Western blot.

Photo-induced crosslinking of UBL probes with proteins contained in cell extracts 1h 50m 20s

28 30m 20s

- Whole cell extract from cells ectopically overexpressing the protein of interest or not can be prepared from any chosen or available cell types (e.g. HEK293T or HeLa cells). Cell lysate can be prepared using lysis buffer, with incubation On ice for 00:20:00 , and centrifugation at 17000 x g, 4°C, 00:10:00 .
- Alternatively, cells can be re-suspended in PBS supplemented with protease inhibitors and lysed by sonication (QSonica Q125 sonicator with a 2 mm diameter probe) On ice for 00:00:20 at 30% amplitude 5 s on/5 s off, followed by centrifugation as above.

Lysis buffer

A	B
Tris-HCl pH 7.5	10 mM
NaCl	150 mM
EDTA	0.5 mM
MgCl2	2 mM
Nonidet P-40	0.5%
Benzonase	2.5 units/µl
c0mplete EDTA-free protease inhibitors (Roche)	1x

Note

N-terminally GFP-tagged N-terminal (1-103) NS1B (Non-structural protein 1 from Influenza B, uniprot ID: P03502 and accession number: NP_056666) was expressed from a pEGFP-C1 background.

29 Incubate the biotinylated BpF-containing UBL (final concentration [M] 20 micromolar (µM)) and 2.5 undetermined - 8 undetermined (final concentration) whole cell extract, in a total volume of 100 µL for 00:15:00 at 4 °C with rotation (e.g. roller or rotating wheel). 15m

Note

The UBL proteins would be in PBS buffer pH 7.4, and the whole cell extract would be in lysis buffer (see above).

30 Divide the sample into two parts. One part is incubated at Room temperature , and the other part is irradiated for 01:00:00 with long-wave UV light (λ=365 nm; Herolab UV-16 L, 6 W, 6 mm distance). 1h

31 After irradiation, add the same volume of 2 × SDS loading buffer to the samples followed by heating for 00:05:00 at 95 °C . The crosslinked band can be analysed by SDS-PAGE and Western blotting. 5m

Enrichment of UBL binding partners from whole cell extract 4h 15m

32 Mix the biotinylated UBL probe ([M] 20 micromolar (µM)) with HEK293T or HeLa cell extract (5 undetermined - 10 undetermined) in PBS buffer supplemented with HALT protease inhibitor cocktail (Thermo Scientific) in a total volume of 100 µL and incubate for 00:15:00 at 4 °C . 15m

33 Divide the sample in half. Irradiate one part for 01:00:00 with long-wave UV light using a hand-held UV lamp (λ=365 nm; Herolab UV-16 L, 6 W, 6 mm distance). 2h

- If necessary, perform this step at 4 °C to avoid overheating of the sample. Incubate the other part without UV irradiation for 01:00:00 at a comparable temperature.

34 Afterwards, add approximately 50 µL streptavidin sepharose beads (Cytiva, 17-5113-01) added to each sample and incubate for 02:00:00 at 4 °C on a rotation wheel. 2h

Note

Wash the beads beforehand 4 times with PBS buffer.

35 Wash the beads thoroughly 5 times with 500 µL of each of the following buffers using spin columns (Pierce Micro-Spin Columns, 10510824):

1. Buffer A

A	B
urea	8 M
SDS	2%
Tris	100 mM
NaCl	200 mM
pH 8	adjusted with HCl

2. Buffer B

A	B
urea	8 M
SDS	0.2%
ethanol	10%
isopropanol	10%
Tris	100 mM
NaCl	1.2 M
pH 8	adjusted with HCl

3. Buffer C

A	B
urea	8 M
SDS	0.2%
ethanol	10%
isopropanol	10%
Tris	100 mM
NaCl	200 mM
pH 5	adjusted with HCl

4. Buffer D

A	B
urea	8 M
SDS	0.2%

A	B
ethanol	10%
isopropanol	10%
Tris	100 mM
NaCl	200 mM
pH 9	adjusted with HCl

Note

All urea buffers should be freshly prepared; buffer A: 00:02:00 incubation time on the beads before centrifugation; buffer B-D: 00:01:00 incubation time.

36 Wash the beads 10 times with 500 µL buffer E (50 undetermined ammonium bicarbonate (NH_4HCO_3)) to ensure that the SDS is washed away properly and to prepare for on-bead or in-gel digestion (see below).

Note

Both on-bead and in-gel trypsin digestion were tested following streptavidin-mediated enrichment of UV-crosslinked proteins. Sample preparation choice depends on the protein of interest and available pipelines.

Mass spectrometry preparation and analysis

21h 9m

37 **On-bead digestion**

37.1

- Transfer the streptavidin resins to a fresh reaction tube with a spatula and add 20 µL of buffer E.
- For an on-bead tryptic digest, add DTT ([M] 5 millimolar (mM) final concentration) [dissolved in freshly prepared 50 undetermined NH_4HCO_3] and incubate for 00:30:00 at 56 °C while shaking.

30m

37.2 Add 2-iodoacetamide (IAA, [M] 25 millimolar (mM) final concentration) [dissolved in freshly prepared [M] 50 millimolar (mM) NH_4HCO_3] and incubate for 00:20:00 at Room temperature in the dark.

20m

37.3 Add DTT ([M] 15 millimolar (mM) final concentration) [quenching of IAA], and then add 200 ng trypsin (e.g. Trypsin Gold, Promega or Sigma) dissolved in 10 µL of [M] 50 millimolar (mM) NH_4HCO_3.

37.4 After 00:10:00 , add 30 µL of ProteaseMax (Promega) in [M] 50 millimolar (mM) NH4HCO3 [0.1%] in water. Incubate the mixture Overnight at 37 °C on a rotation wheel. 20m

37.5 Acidify the supernatant with formic acid (FA), vacuum dry completely and resuspended in 7 µL in acetonitrile/water (2% v/v), acidify with FA (0.1% v/v) and analyse by LC-MS/MS.

Note

1 µL for each technical repeat is used.

38 **In-gel digestion**

38.1
- After washing, material was eluted by boiling in SDS sample buffer and loaded on a homemade 15% SDS-PAGE gel.
- Gel lanes were sliced into 5 mm horizontal slices and placed into separate 1.5 ml low bind Eppendorf tubes.

38.2 Gel slices were dehydrated with 900 µL HPLC grade acetonitrile and incubation at Room temperature for 00:15:00 with agitation (e.g. thermomixer at 900 rpm). 30m
- The supernatant was removed before rehydration with 900 µl HPLC grade water and incubation at Room temperature for 00:15:00 with agitation.
- This cycle of dehydration-rehydration was repeated a total of 5 times to ensure efficient removal of both aqueous and organic solvent soluble contaminants.

38.3 The gel pieces were dehydrated a final time with 900 µL HPLC grade acetonitrile and incubated as above. The supernatant was aspirated off and gel slices were dried to completeness in a vacuum centrifuge at 45 °C for 00:30:00 . 30m

38.4 Gel slices were rehydrated in 40 µL [M] 50 millimolar (mM) TEAB (#catT7408, Sigma) and sequencing grade trypsin (source as above) at a concentration of 20 undetermined . 18h 20m
- Gel slices were incubated without shaking at Room temperature for 00:20:00 before the addition of 100 µL [M] 50 millimolar (mM) TEAB and incubation at 37 °C with shaking (e.g. using a thermomixer) at 900 rpm, 18:00:00 .

38.5 Overnight digests were acidified by the addition of formic acid to a final concentration of 0.2% (v/v).

- Digest supernatants from the same lane were pooled together and peptides dried to completeness in a vacuum centrifuge at 45 °C .
- Peptide digests were finally resuspended in 5 µL 0.2% formic acid immediately prior to LC-MS/MS analysis.

39 **LC-MS/MS**

LC-MS/MS was performed with use of a Vanquish Neo HPLC system coupled to an Orbitrap Lumos mass spectrometer via an EasySpray source (Thermo). The HPLC utilised mobile phases A (0.1% formic acid in water) and mobile phase B (80% acetonitrile, 0.1% formic acid).

40 39m

- Peptides were injected directly onto an Ionopticks reverse phase Aurora TS column (25 cm long, 75 µm ID) using a pressure/flow control protocol utilising a maximum pressure of 800 bar or 600 nl/min.
- Peptides were separated at 180 nl/min with a gradient of 1-4% B over 00:01:00 followed by 4-40% B% over 00:38:00 .
- LC eluent was sprayed directly into the MS source at an ion spray voltage of 1.7 Kv.
- The MS was operated in data independent acquisition (DIA) mode.
- An MS1 orbitrap scan was performed at 120K resolution with an m/z range of 350-1200 with a target value set to standard mode and injection time set to auto.
- Targeted orbitrap MS2 scans were set at 361.7, 384.1, 406.84, 428.8, 451.2, 473.5, 495.9, 518.3, 540.6, 563.0, 585.4, 607.7, 630.1, 652.5, 674.8, 697.2, 719.6, 719.9, 764.3, 786.7, 809.1, 831.4 and 853.8 using HCD normalised collision energy of 30% set to z 2 and a mass range of 23.5 Th.
- The Orbitrap MS2 scans were performed at a resolution of 15K (at m/z 200) with a normalised AGC target of 2000% and a max fill time of 22 ms.
- DIA data was processed using Spectronaut 17 (Biognosys) in direct DIA mode using oxidation (M), deamidation (NQ) and Acetylation (N term) as variable modifications in a search against a human Uniprot database.
