# Supplementary figures and images for: Site-selective photo-crosslinking for the characterisation of transient ubiquitin-like protein-protein interactions
Source: PLoS One. 2025 Jan 27;20(1):e0316321. doi: 10.1371/journal.pone.0316321 (PMC11771908; doi:10.1371/journal.pone.0316321)

Figure 3B

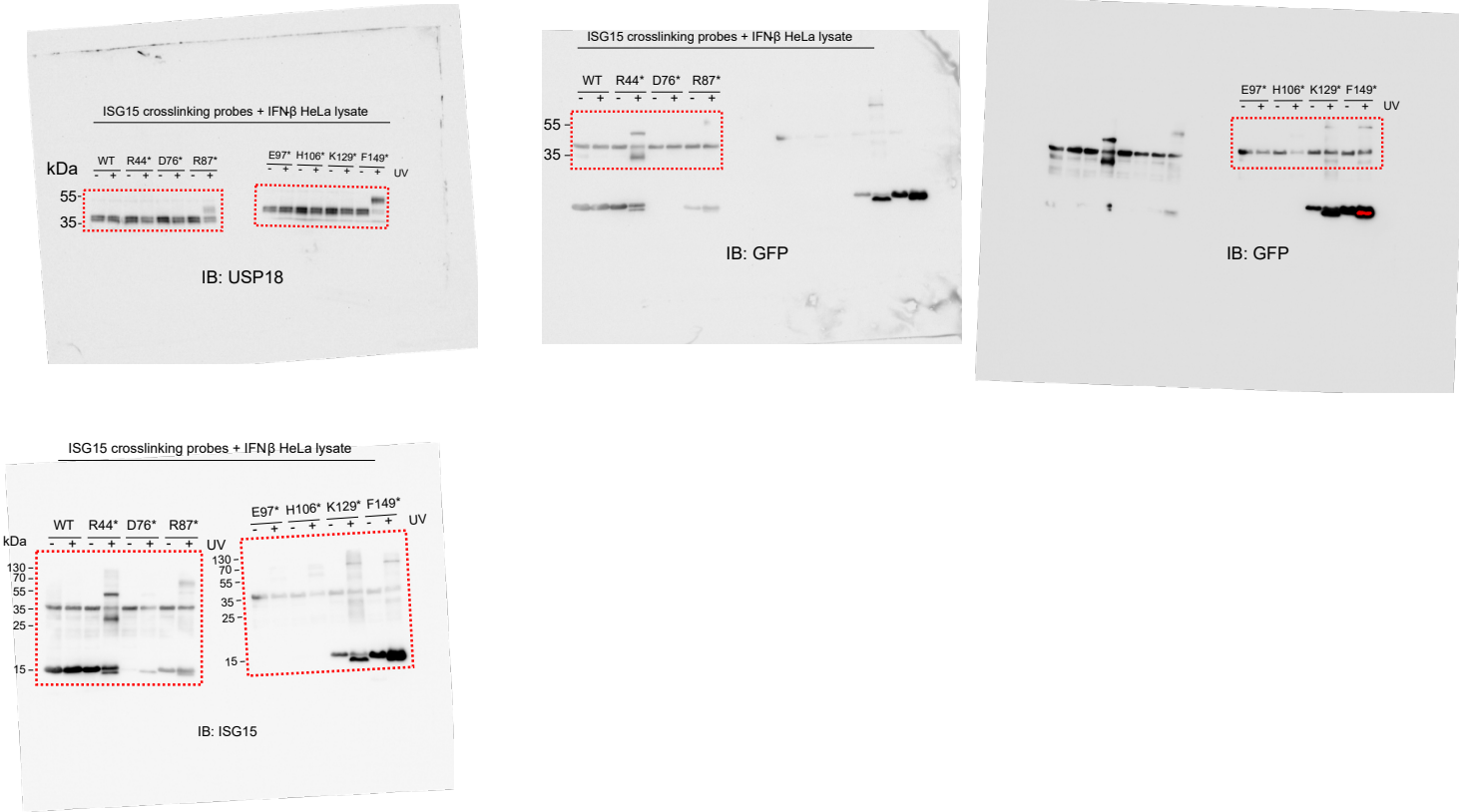

Figure 4A

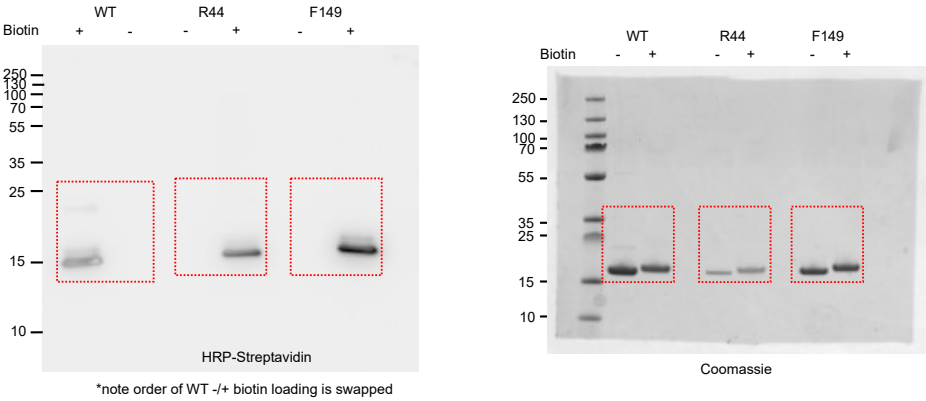

Figure 4B

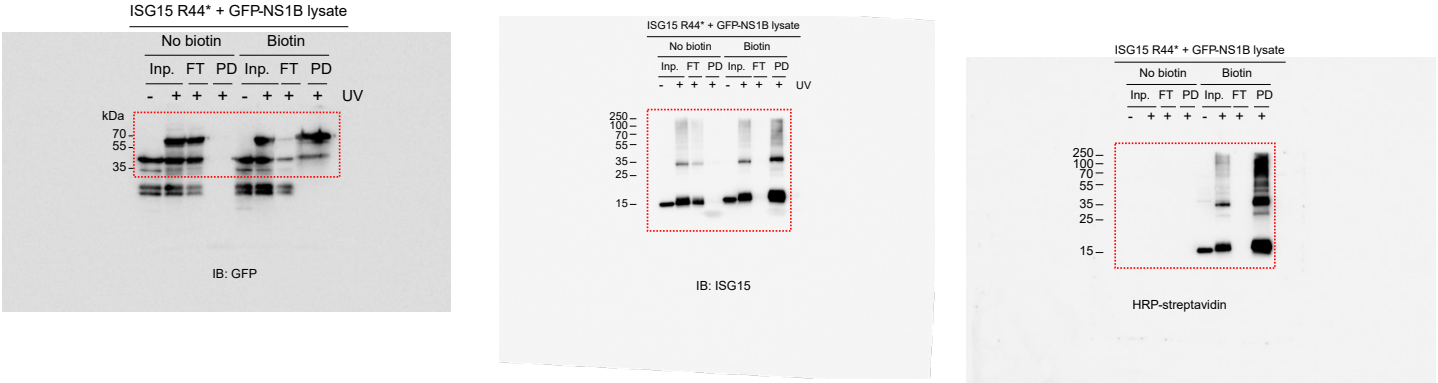

Supplement: S1 Raw images — (PDF) [file pone.0316321.s002.pdf]
